# Novel Approach for Synthesis of Graphene-like Phases by Pulsed Laser Ablation in a Flow-Mode Suspension

**DOI:** 10.3390/ma15227870

**Published:** 2022-11-08

**Authors:** Ivalina Avramova, Dimitar A. Dimov, Nadya Stankova, Miroslav Petrov, Desislava Karaivanova, Georgi Avdeev, Stoyan Russev, Daniela Karashanova, Biliana Georgieva, Evgeniya Valcheva, Teodor Milenov

**Affiliations:** 1 Institute of General and Inorganic Chemistry, Bulgarian Academy of Sciences, Acad. G. Bonchev Street, Bl. 11, 1113 Sofia, Bulgaria; 2 “Academician Emil Djakov” Institute of Electronics, Bulgarian Academy of Sciences, 72 Tzarigradsko Chaussee Blvd., 1784 Sofia, Bulgaria; 3 “Academician.Rostislav Kaishev” Institute of Physical Chemistry, Bulgarian Academy of Sciences, Acad. G. Bonchev Street, Bl. 11, 1113 Sofia, Bulgaria; 4 Faculty of Physics, University of Sofia, 5 James Bourchier Blvd., 1164 Sofia, Bulgaria; 5 “AcademicianJordan Malinowski” Institute of Optical Materials and Technologies, Bulgarian Academy of Sciences, Acad. G. Bonchev Street, Bl. 109, 1113 Sofia, Bulgaria

**Keywords:** pulsed laser ablation in liquid, method, characterization, graphene, graphene oxide, reduced graphene oxide

## Abstract

The present study investigates the possibility of obtaining graphene-like phases (defected graphene, graphene oxide, and reduced graphene oxide) as fine suspensions by applying a novel pulsed laser ablation (PLA) approach in flow mode. Two types of suspensions of microcrystalline graphite in aqueous suspensions and two types of microcrystalline graphite in suspensions of 6% hydrogen peroxide solution were irradiated in a quartz tube through which they flow. The third (λ = 355 nm) and fourth harmonics (λ = 266 nm) of an Nd:YAG laser system (15 ns pulse duration and 10 Hz pulse repetition rate) were used. The morphology of the obtained particles was studied by transmission electron microscopy (TEM). Their phase composition and structure were explored by X-ray photoelectron spectroscopy, X-ray diffractometry, and Raman spectroscopy.

## 1. Introduction

Pulsed Laser Ablation in Liquid (PLAL) has been proven as a simple and effective technique to fabricate the different types of carbon-related nanomaterials including nanodiamonds, few-layer to multilayer graphene nanosheets, and graphene oxide nanosheets [1]. Systemic improvements in PLAL design, controlled laser ablation parameters (wavelength, pulse width, repetition rate, optics of laser beam, and fluence), and the appropriate selection of the carbon target and liquid environments have resulted in the formation of a wide range of carbon nanomaterials such as nanodiamonds [2], carbogenic nanoparticles, bilayers of the few-layer graphene sheet [3], carbon nanotubes, graphene oxide, reduced graphene oxide, graphene and graphene oxide quantum dots, polyynes, and carbon-encapsulating metal nanoparticles [4,5]. PLAL techniques have also been explored for the synthesis of fluorescent carbon nanomaterials [6,7].

The use of PLAL for the synthesis of carbon materials is chemically and technically very simple. Almost no byproducts are created and no catalyst is used. Many sources of carbon can be used as the starting material.

There are three main types of PLAL techniques used to synthesize carbon nanomaterials.

The first experimental design of PLAL included a focused laser beam directed on the surface of a solid target immersed in a liquid for generating the colloidal carbon nanomaterials: nanodiamonds and quantum dots [8,9], as well as graphene-like phases (graphene, defected graphene, reduced graphene oxide (rGO), and graphene oxide (GO) [10]. This PLAL technique is known as the batch method.

Further on, the solid target is replaced with a suspension (e.g., of micrographite particles in bidistilled water), which significantly increases the useful yield in the form of stable suspensions of nano-sized carbon phases in water (see, for example, [11]). The latter method (regardless of whether focused or defocused light is used, as well as the direction of irradiation of the suspension beaker) is usually defined as the semi-batch method. 

Another implementation of PLAL uses a jet stream of a suspension of carbon particles irradiated by a focused laser beam [12]. The colloidal solution can be circulated in a closed loop for continuous irradiation [13]. This method of synthesizing carbon nanomaterials is known as the flow jet method. In the new approach we report herein, which we can also determine as a PLA in a continuous flow system (flow-mode PLA method), the irradiation is performed in a quartz tube, through which the suspension flows under the action of gravity. The main differences between our proposed flow mode method and the flow jet method are the irradiation inside the quartz tube, which ensures the stability of the flow; the lack of contact with the environment, which allows for efficient functionalization; and the low flow rate, which enables the complete irradiation of the suspension. PLA in flow mode can be implemented in several variants depending on the type of fluid flow, laser-beam focusing, and the location of irradiation.

The present research examines the opportunity of obtaining graphene-like phases (functionalized defected graphene, graphene oxide, and reduced graphene oxide) and studies their modification and oxidation level when applying a pulsed laser ablation in liquid (PLAL) in flow mode.

The phase composition of the obtained sedimented suspensions was characterized by different methods: X-ray photoelectron spectroscopy (XPS), grazing incidence X-ray diffractometry (GIXRD), Raman spectroscopy, and transmission electron microscopy (TEM)

## 2. Materials and Methods

Two different types of suspensions based on two graphites, 99.9995% purity Ultra F (Alfa Aesar, Haverhill, MA, USA), designated as HQ, and low-grade graphite (TEOCOM Ltd., Sofia, Bulgaria), designated as LQ, which were obtained by mixing 0.4 g of graphite in 100 mL of bidistilled water. Hydrogen peroxide solution (100 mL 6% H_2_O_2_ in bidistilled water) was used in the other suspension as the liquid medium, maintaining a concentration of 4 mg/mL solid to liquid in all experiments of this study. The suspensions were prepared and placed in an ultrasonic bath 30 min before irradiation.

**Figure 1:**
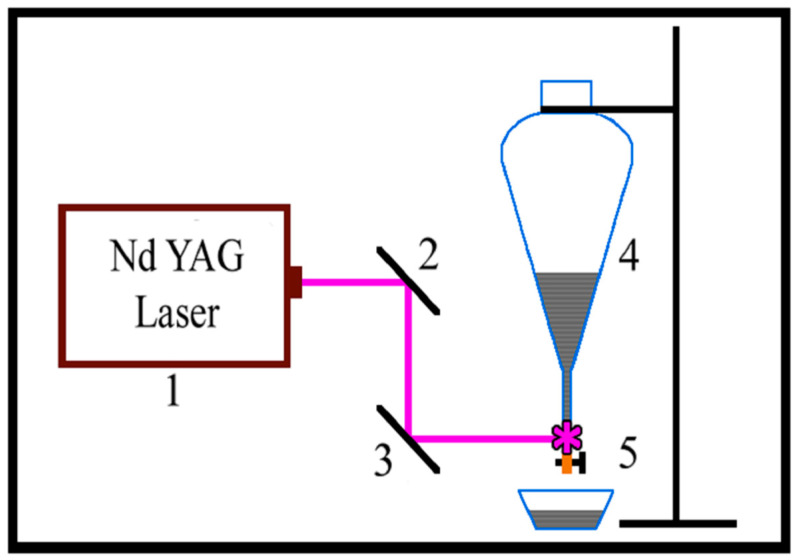
Schematic diagram of the experimental setup: 1—Nd: YAG laser; 2 and 3—Mirrors; 4—Dropping funnel; 5—Dosing quartz tube.

The system that we used is shown in Figure 1 and consists of an Nd:YAG laser—1, mirrors—2 and 3, a reservoir for graphite suspension—4, and flow-through elements. i.e., a silicone tube and an end quartz tube with an inner diameter of 5.5 mm and wall thickness of up to 0.8–1.0 mm. The third and fourth (λ = 266 nm) harmonics of the Nd:YAG laser (λ = 1064 nm fundamental wavelength, 15 ns pulse duration, 10 Hz pulse repetition rate) were λ = 355 nm and λ = 266 nm, respectively. The 0.364 J/cm^2^ and 0.465 J/cm^2^ laser beam fluencies for PLA with λ = 266 nm and 355 nm, respectively, were used in different experiments. The irradiation of graphite suspensions was conducted while they gravitationally forced flow through the quartz tube (see Figure 1). An unfocused laser beam with a spot 5.5 mm wide and 4.5 mm high was used, which exactly fit the internal diameter of the quartz tube.

The dispenser placed after the irradiation zone allowed for the adjustment of the flow rate so that the quartz tube was always full without flow turbulence and thus the maximum amount of irradiated suspension was ensured. The absorption spectra of water and hydrogen peroxide in the UV electromagnetic wave region showed the absorption maxima at a wavelength of λ = 250 nm and an absorption minimum at a wavelength of λ = 350 nm, whereas the absorption of graphite at these two wavelengths was close. Our chosen wavelengths of laser radiation (λ = 266 nm and λ = 355 nm) were close to the maximum and minimum absorption values shown; therefore, the radiation was absorbed by all phases of the suspension at λ = 266 nm and mainly by the graphite phase at λ = 355 nm. 

The irradiation of the entire suspension lasted about 15 min. The obtained graphene-like phases were about 8–11% of the mass of the starting graphites, but we must note that part of the modified particles agglomerated with the main phase and settled. Irradiation took place in an environment isolated from atmospheric air, which, combined with a suitable liquid medium, enabled not only the preparation of different carbon phases but also the realization of their effective functionalization, which is an advantage over other PLAL methods. For the implementation of this method of laser ablation in liquid, no complicated equipment is needed, and there are no moving parts. This makes the PLAL in flow mode method highly reproducible and very easy to apply.

The Raman measurements were carried out using backscattering geometry with a micro-Raman HORIBA Jobin Yvon Labram HR 800 visible spectrometer equipped with a Peltier-cooled CCD detector with a He-Ne (633 nm wavelength and 0.5 mW) laser excitation. The laser beam was focused on a spot of about 1 μm in diameter, the spectral resolution being 0.5 cm^−1^ or better. The X-ray photoelectron spectroscopy (XPS) analyses were carried out using an ESCALAB MKII spectrometer with a non-monochromatic Al X-ray source under vacuum greater than 10^−8^ Pa at a 45° take-off angle. The Au4f photoelectron line was used for the calibration of the spectra. The accuracy of the measured BE was 0.2 eV. The photoelectron lines of the constituent elements on the surface were recorded and corrected by subtracting a Shirley-type background and quantified using the peak area and Scofield’s photoionization cross-sections. The C1s, O1s, and Auger lines were recorded for each specimen and further on, the C1s lines were subjected to an additional fitting procedure with XPSPEAK 4.1 software.

The structure of the suspensions after drying was characterized by XRD using a PANalytical Empyrean apparatus, whereas the local morphology and structure were revealed by a high-resolution transmission electron microscopy (HR TEM) characterization in both high-resolution (HR) and selected area electron diffraction (SAED) modes with an HR STEM JEOL JEM 2100 microscope.

## 3. Results

There is always an amount (20–50% from initial powders) of unmodified graphite particles in the resulting suspensions, which settle up to 5 min after the laser treatment and are removed from the remaining suspension by decantation. The suspension obtained after decantation was sufficiently stable over time: visible precipitation occurred after more than 24 h. For further research, powders obtained after drying the decanted suspension at 50 °C were used. In a control experiment, suspensions prepared in the manner described were examined without being irradiated and showed results consistent with those of pristine graphite.

### 3.1. XPS Characterization

XPS analysis is useful for identifying the chemical composition, hybridization (sp^2^ and sp^3^), structure, and defects (edges, functional groups, pentagon, and heptagon) of carbon materials [14].

Suspensions from two types of graphite named HQ graphite and LQ graphite, with different degrees of purity before and after laser treatment with 266 nm and 355 nm wavelengths in distilled water and distilled water with 6% hydrogen peroxide were investigated using X-ray photoelectron spectroscopy. The C1s photoelectron spectra were used for the evaluation of the type of carbon hybridization, as well as their Auger spectra. The obtained photoelectron spectra had a complex shape due to the existence of different bonds on the surface of the studied suspensions. In order to evaluate the type of hybridization, the sp^3^/sp^2^ ratio was calculated as well as an evaluation of the existence of other types of bonds in the resulting suspensions, whose components the C1s were fitted to. 

The constituent elements on HQ graphite and LQ graphite surfaces were evaluated and the respective data are presented in Table 1.

The formation of defects on the surface and edges of HQ graphite was decisive for the degree of oxidation, for which high photon energy is required. Therefore, the amount of oxygen as a result of irradiation at λ = 266 nm was higher than the amount of oxygen as a result of irradiation at λ = 355 nm, where the photon energy was lower and fewer defects were formed.

On the surface of graphite LQ, defects already existed and laser-induced oxidation processes played a leading role in oxidation. In this case, the amount of oxygen increased as the energy flow increased, which at λ = 266 nm, was 0.364 J/cm^2^, and at λ = 355 nm, it increased to 0.465 J/cm^2^. PLAL in flow mode allowed such results to be obtained for different materials, different parameters of suspensions, and laser radiation and on that basis, to carry out controlled and efficient functionalization with oxygen and other atoms.

**Figure 2:**
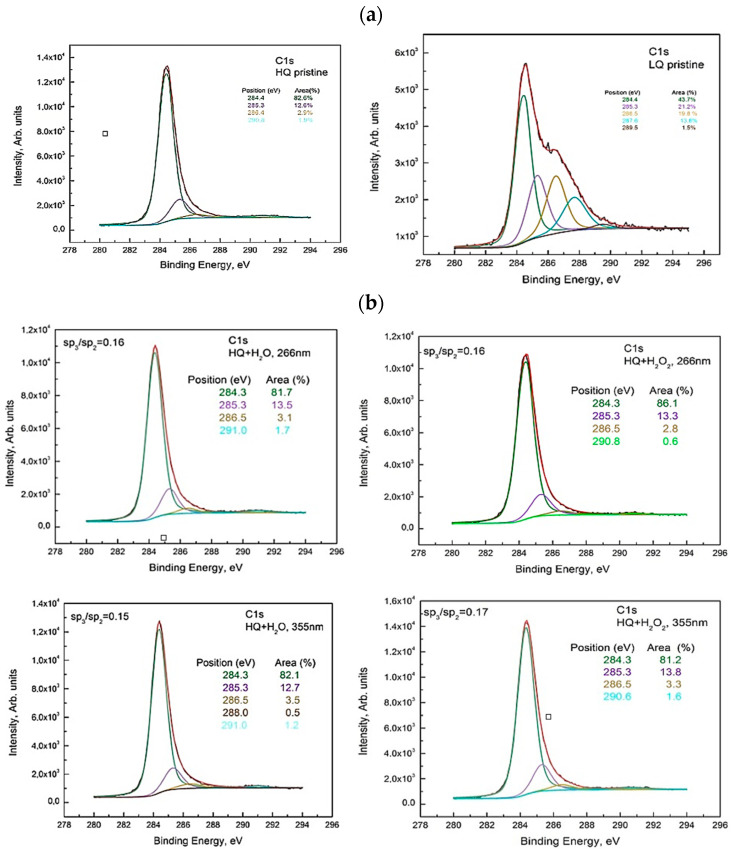
Deconvolution of the C1s line of the XPS spectrum taken from (**a**) HQ pristine and LQ pristine (**b**) HQ+H_2_O and HQ+H_2_O_2_ sediment for both wavelengths 266 nm and 355 nm.

**Figure 3:**
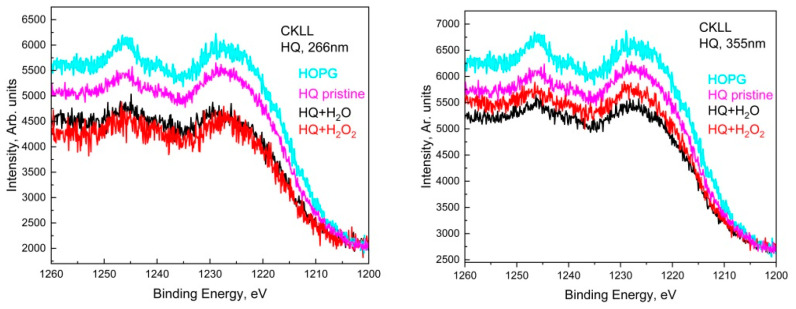
Auger spectra of HQ+H_2_O and HQ+H_2_O_2_ sediments for both wavelengths, 266 nm and 355 nm, compared to HQ pristine and HOPG.

The deconvolution spectra of C1s for HQ pristine, as well as C1s for the suspensions from HQ graphite, showed several peaks at around 284.4 eV, 285.3 eV, 286.5 eV, and 291.0 eV attributed to the non-oxygenous carbon skeleton (sp^2^-hybridized carbon structure), sp^3^-, hydroxyl C–OH/epoxy groups, C–O–C functional groups, and π-π* bond. The C–H bond and oxygen-containing bonds were formed due to the presence of edge and defect sites in the graphite, which provided suitable anchoring sites for the functionalities [15]. The peak at 284.4 eV describes the sp^2^-hybridized C atoms arranged on the corner of a hexagon as in graphite. The peak at ∼285.4 eV corresponds to sp^3^-hybridized carbon atoms and likely originated from unsaturated carbon atoms reacting with hydrogen during the oxidation process. An increase in the oxygen content on the surface of HQ graphite was observed after laser treatments in H_2_O, as well as in H_2_O + 6% H_2_O_2_ for both laser wavelengths compared to the pristine one. The calculated sp^3^/sp^2^ ratio did not change drastically (see Figure 2a,b). This observation was confirmed by a comparison of the Auger CKVV line of the studied suspensions with that of highly oriented pyrolytic graphite (HOPG) (see explanation below and Figure 3).

The results obtained for the second type of graphite, denoted as LQ graphite, treated in the same solutions and wavelengths as the laser, were completely different.

**Figure 4:**
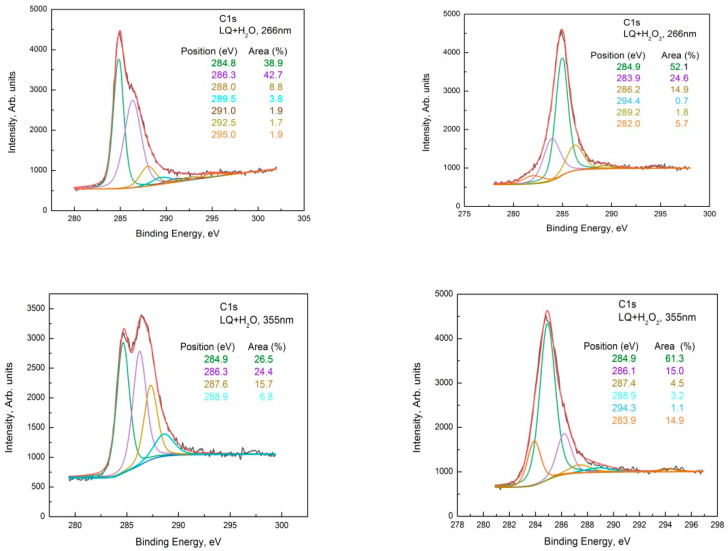
Deconvolution of the C1s line of the XPS spectrum taken from the LQ pristine, LQ+H_2_O, and LQ+H_2_O_2_ sediments for both wavelengths, 266 nm and 355 nm.

The C1s photoelectron spectra of LQ+H_2_O and LQ+H_2_O_2_ treated at both wavelengths are shown in Figure 4. The C1s of LQ pristine are shown in Figure 2a, and the sp^2^, sp^3^, C-O, and C=O, as well as the O-C=O groups, were observed on its surface. The C1s of LQ graphite treated in H_2_O with a 266 nm wavelength were fitted with seven peaks situated at around 284.8 eV, 286.3 eV, 288.0 eV, 289.5 eV, 291.0 eV, 292.5 eV, and 295.0 eV, assigned to the C-C, C-O, C=O, O-C=O, and π-π* satellite groups, respectively, of the graphene oxide phase. This shows that during the oxidation reaction, various functional groups, such as hydroxyl and epoxide that were randomly distributed on the basal surface and carboxylic acid at the edges, were introduced, [16] which intercalated with water molecules to increase the interlayer spacing. Previously, it was proved that the enlarged layer distance made it possible to delaminate graphite oxide into graphene oxide (GO) under ultrasonication [17]. The presence of oxygen functionalization in GO partially breaks sp^2^−sp^2^ bonds into sp^3^−sp^3^ bonds. 

Additionally, in the spectra appeared shake-up satellite peaks in comparison with the pristine one in the high-binding energy range (higher than 292.0 eV). The peaks that appeared at higher-binding energy regions can be associated with the formation of C_60_ and carbon nanotubes (CNT) [18].

**Figure 5:**
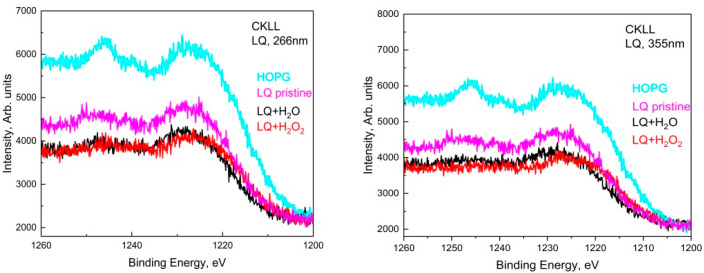
Auger spectra of LQ+H_2_O and LQ+H_2_O_2_ sediments for both wavelengths, 266 nm and 355 nm, compared to LQ pristine and HOPG.

Similarly, in the deconvoluted C1s spectra of LQ graphite treated in H_2_O_2_ with 266 nm, seven peaks acted, situated at around 284.8 eV, 286.3 eV, 288.0 eV, 289.5 eV, 291.0 eV, 295.0 eV. In addition, two other peaks appeared at around 282.0 eV and 283.9 eV. The shoulder appeared at around 282.0 eV, most probably due to a charge-neutralized fraction due to the existence of hydrocarbon [19], whereas the other one was due to the C=C bonds. It seems that subsequent GO transformations into reduced GO (rGO) took place and the π-conjugated structure was restored, as well as the conductivity resembling graphene. The 355 nm laser treatment of LQ graphite suspensions in H_2_O and H_2_O_2_ caused similar changes in the C1s photoelectron line. Only four peaks were used for the fitting of C1s to H_2_O; however, seven peaks were used for the fitting of C1s to LQ graphite in the H_2_O_2_ suspension. Again, the laser treatments of H_2_O led to the formation of GO, and the same laser treatment of LQ graphite in H_2_O_2_ led to the formation of rGO. Furthermore, the peaks that appeared higher than 292.0 eV in the C1s spectra were associated with the formation of C_60_ and CNT [18]. CKVV Auger spectroscopy is a more suitable technique for the identification of the chemical state of carbon atoms on the uppermost layers because the Auger line shape is a probe of the valence band electronic structure [20]. The CKVV Auger spectrum can also be used as an indication of the existence of sp^2^ or sp^3^ features in carbon-like materials [21]. The slopes of the right parts of the obtained spectra for the pristine laser-treated graphite in comparison with that of HOPG were obviously different. The slope of the pristine HOPG was typical for sp^2^ bonds, whereas the slopes of the spectra for the other samples gradually changed and tended to that of the sp^3^ bond, especially for the laser-treated suspensions prepared with LQ graphite (see Figure 5).

### 3.2. Raman Spectroscopy

**Figure 6:**
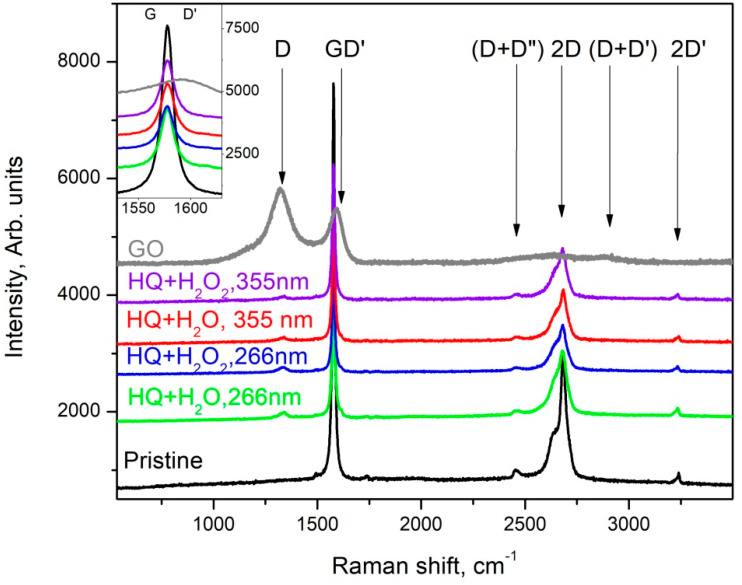
Raman spectra taken from different flakes of the sediment from the suspension HQ of microcrystalline graphite.

**Figure 7:**
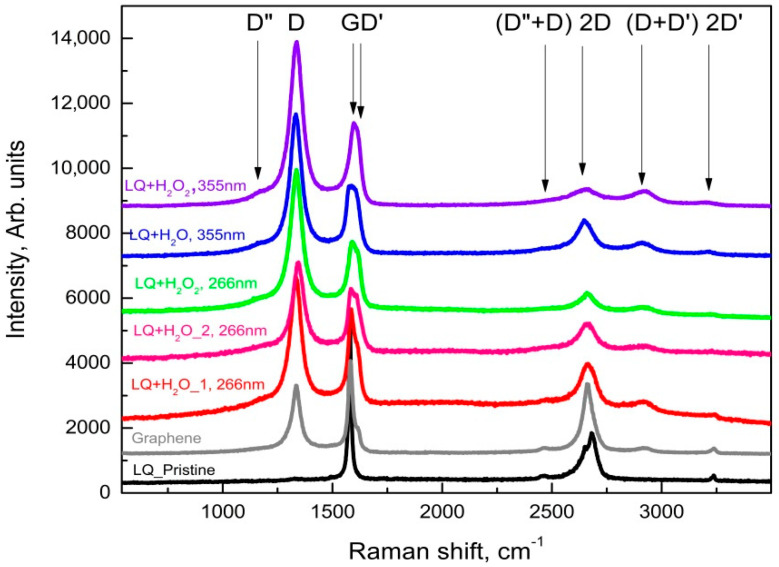
Raman spectra taken from different flakes of the sediment from the suspension LQ of microcrystalline graphite.

It is well known that Raman spectroscopy is a very powerful tool in the characterization of carbon materials, e.g., it was found that the Raman spectrum of graphene is its fingerprint [22] and its careful analysis allows for the determination of the number of layers in the studied samples; the ratio of sp^2^/sp^3^ hybridized carbon can be determined [23]; and graphene oxide (GO) and reduced graphene oxide (rGO) can also be distinguished according to their Raman spectra [24]. The results of the Raman spectroscopy characterization are summarized in Figure 6 and Figure 7, where the Raman spectra of the main phases observed in the dried suspensions after the various PLA treatments are shown.

The main phase in the suspensions of HQ graphite after PLA treatment is graphite microcrystals with increasing defects; as the D-band appears (at about 1331 cm^−1^), the intensity of the G- and 2D bands decreases. It should also be noted the formation of:

- a very fine crystalline (or semi-amorphous) phase of GO (gray trace in Figure 6) when treating suspensions of HQ graphite in bidistilled water at λ = 266 nm. The largest number of such phases was found in suspensions of HQ graphite in bidistilled water with 6% H_2_O_2_, modified by laser irradiation with a fluence of 0.364 J/cm^2^;
- a lot of graphenes, as well as defected graphene flakes, which were observed in all suspensions irradiated with a λ = 266 nm wavelength. Such objects were observed significantly less frequently in suspensions modified by a λ = 355 nm wavelength laser irradiation. Their Raman spectra were quite similar to those presented in Figure 7.

In contrast to the suspensions discussed above, a large variety of graphene-like phases (graphene, defected graphene, rGO, and GO) were observed in those of LQ graphite in bidistilled water and bidistilled water with 6% H_2_O_2_. It should be noted that in these suspensions after their treatment with laser irradiation, graphite microcrystals were rarely found. The main phases in these suspensions were defected graphene (the red trace in Figure 6) [25], reduced graphene oxide (the pink line in Figure 7), and GO (Raman spectrum similar to the gray trace in Figure 6). It is noteworthy that the addition of H_2_O_2_ to the suspensions increased the intensity of the D’ and (D+D’) bands (see the pink trace denoted by LQ+H_2_O_2 at 266 nm, as well as those denoted by LQ+H_2_O_2_ at 266 nm, LQ+H_2_O at 355 nm, and LQ+H_2_O_2_ at 355 nm), which were associated with the enrichment of graphene/graphite phases with oxygen-containing radicals [19]. Similar to PLA experiments on HQ graphite suspensions, all treated suspensions of G graphite can be isolated particles, with Raman spectrum corresponding to single- to few-layered graphene (gray line in Figure 6): the FWHM of its 2D band was between 45 and 65 cm^−1^, whereas the intensity of the 2D band was comparable to that of the G-band. It should be noted that such flakes were observed more frequently in suspensions irradiated with a 355 nm laser wavelength.

### 3.3. XRD Investigations

**Figure 8:**
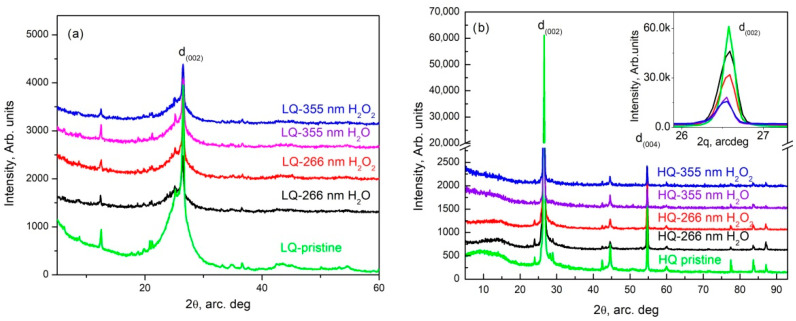
XRD pattern of LQ (**a**) and HQ (**b**) specimens.

The XRD patterns obtained from pristine HQ graphite (Figure 8b—green trace) completely coincided with those shown by M.S. Seehra et al. [26] and show that the analyzed sample consisted of hexagonal—(see COD # 96-101-1061) and rhombohedral (ICSD #31829) graphite. The only peaks that did not belong to these phases were the low-intensity peaks at 2θ values at around 28 and at 29 arcdeg, which coincided completely with the peaks of fullerene C60 (COD # 96-800-0216). It should be noted that the stronger peaks of LQ graphite also coincided with the indicated phases.

The d_(002)_ graphite reflection (COD # 96-101-1061) intensity decreased several times (more than eight times) after the PLA process using 355 nm and fluence of 0.465 J/cm^2^ in HQ graphite suspensions. Moreover, the addition of 6% H_2_O_2_ to the initial suspension of graphite HQ in water further reduced the peak by more than 50%. In parallel, the same peak practically did not change its position but significantly increased its full width at a half maximum (FWHM). The increase in this parameter in those treated with radiation with a wavelength of 266 nm was more than 50% in both suspensions. The same parameter (FWHM of d_(002)_ reflection) determined in the XRD patterns of the suspensions modified with a wavelength of 355 nm increased by 35% in the suspension in bidistilled water and by 63% in that in a solution of hydrogen peroxide in bidistilled water. The above results undoubtedly show that PLA processes cause fragmentation of graphite particles, which most likely takes place by the exfoliation of single- to multilayer graphene and its further agglomeration during sedimentation. The treatment increased the width of all reflections, whereas some reflections disappeared. This allowed us to conclude that a process of amorphization was taking place. 

For LQ graphite, there was a decrease in the reflection intensity and an increase in the width after the PLA process. The detailed analysis of these results did not differ significantly from that already conducted for the 266 nm- and 355 nm-modified suspensions of the same material in water in a non-flow system [11]. By analogy, we can conclude that the studied specimens consisted of mixes of different carbon phases including graphite, fullerenes, carbon nanotubes and graphite intercalation, and exfoliation-induced carbon poly-types, whereas the quantity of graphite significantly decreased.

### 3.4. TEM Characterization

The specimens for the TEM studies were prepared by dropping and drying a drop of any suspension obtained on formvar-covered TEM grids. It should be emphasized that due to the method of obtaining the samples, the visualized nano-sized objects were stuck to each other despite the sonication of the suspension prior to the immobilization on the grid Thus, agglomerates of a much larger size were formed, and individual single-layer graphene flakes were very rarely observed. 

**Figure 9:**
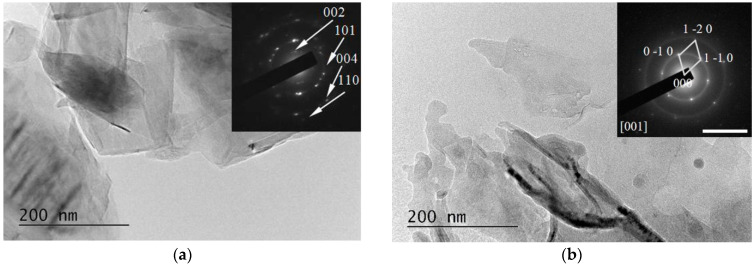
TEM image from an LQ graphite suspension in H_2_O before (**a**) and after (**b**) treatment with λ = 266 nm irradiation. Inset: Selected Area Electron Diffraction (SAED) patterns taken from the central area of the TEM image. The scale bar corresponds to 10 1/nm.

**Figure 10:**
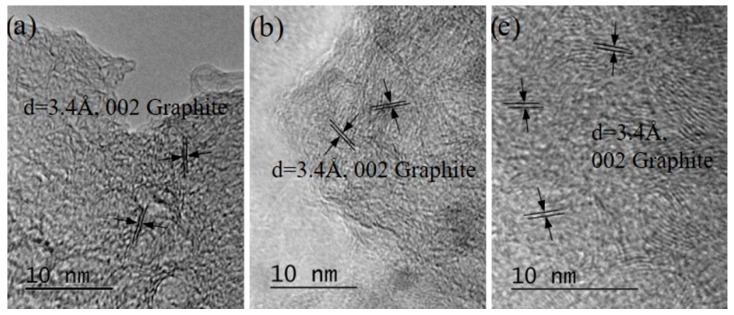
(**a**) HRTEM image of bundles of 3–7 layered graphene/defected graphene obtained by PLAL (λ = 266 nm irradiation) of a suspension of LQ graphite in 6% H_2_O_2_ in bidistilled water. (**b**) HRTEM image of bundles of 1–5 layered graphene/defected graphene obtained by PLAL (λ = 355 nm irradiation) of a suspension of HQ graphite in 6%H_2_O_2_ in bidistilled water. (**c**) HRTEM image of bundles of 3–5 layered graphene/defected graphene obtained by PLAL (λ = 266 nm irradiation) of a suspension of LQ graphite in 6% H_2_O_2_ in bidistilled water.

**Figure 11:**
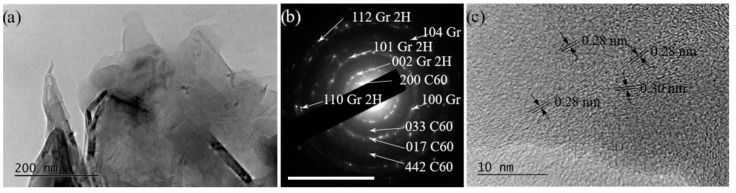
(**a**) An overview TEM image of LQ graphite irradiated with λ = 266 nm H_2_O, demonstrating a complex formation of few-layered graphene/defected graphene flakes agglomerated with 3D nanoparticles of C_60_ fullerenes. (**b**) The SEAD pattern indicating the presence of the reflexes of hexagonal graphite and those of C_60_. The scale bar corresponds to 10 1/nm. (**c**) HRTEM of nano-sized 3D particles of C_60_ with interplanar spacing varying between 0.28 and 0.30 nm.

**Figure 12:**
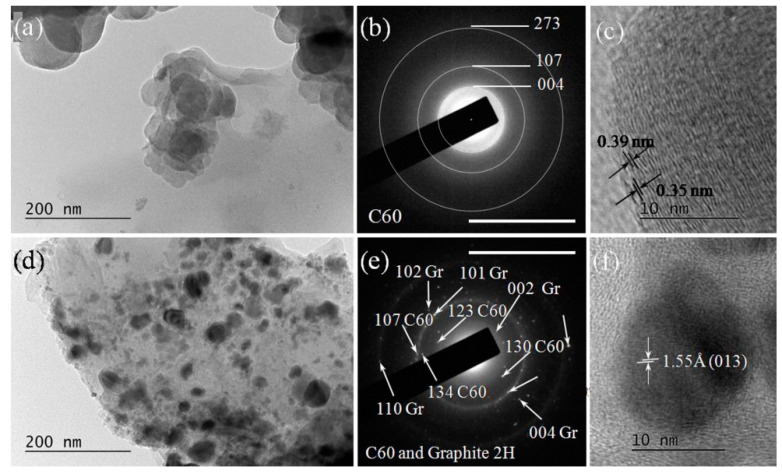
(**a**) Spherically agglomerated amorphized carbon after a 266 nm laser irradiation of HQ graphite suspension in bidistilled water and the corresponding SAED image indicating the fullerene C_60_ (COD- COD # 96-800-0216) (**b**) panel. The scale bar corresponds to 10 1/nm. (**c**) HRTEM image of few-layered fullerene C_60_ sedimented on spheroid amorphous carbon particle. (**d**) Irregularly shaped amorphous carbon particles on which 2D graphene-like flakes and 3D nanoparticles of hexagonal graphite (**f**) were deposited, and the corresponding SAED image (**e**). Images (**d**–**f**) were taken from sediments of the suspension of HQ graphite in bidistilled water irradiated with a 266 nm laser beam.

All the examined suspensions contained microcrystals of graphite. Their quantity was higher in the HQ graphite sample than the LQ graphite sample according to the specifications of the producer for the crystallinity of the two types of graphite samples. An investigation of the particles’ crystalline states using the diffraction mode of the microscope demonstrated the presence of an amorphous mass in the HQ graphite treated with a solution of 6% H_2_O_2_ in water, which probably caused a decrease in the crystals after treatment with the H_2_O_2_ solution. Amorphous material was detected in the LQ graphite sample even before the H_2_O_2_ treatment. Additionally, the TEM characterization of all the suspensions studied showed the formation of different phases after the PLA processes depending on the initial graphite and the composition of the liquid phase of the suspensions:

(i) Graphene and defected graphene flakes, (see, for example, Figure 9). Similar objects were observed in all the studied specimens but were most frequently found in specimens from suspensions treated with 266 nm irradiation and especially in those in 6% H_2_O_2_ in water solutions. The graphene/graphene-like flakes were usually stuck to each other.

(ii) Bundles of 2D (mainly graphene and graphene-like flakes stuck together with fullerene C60), as shown in Figure 10a–c). These bundles consisted of few-layered (2–10 layers) graphene/graphene-like flakes, which were usually plicated or even folded. Such formations were observed in HQ graphite suspensions in 6% H_2_O_2_ in water solutions, as well as more frequently in suspensions of LQ graphite. Aggregates of C60 were observed in all suspensions.

(iii) Sedimented agglomerations of amorphized carbon phases surrounded by 2D (graphene and graphene-like flakes or C60 fullerenes arranged in 2–3 layered nanostructures), as well as 3D (carbon nanoparticles up to 10 nm in size or fullerenes C60), nano-sized particles (see Figure 11 and Figure 12).

The amorphized carbon fraction usually formed small spherically shaped particles with a diameter between 50 and 150 nm and an amorphous structure, which may be often related to the disordered C_60_ fullerene (COD # 96-800-0216) (see Figure 12a). The diffusely blurred polycrystalline SEAD rings (see Figure 12b) corresponded to the interplanar distances of fullerene C60 (COD # 96-800-0216), whereas those in Figure 12e corresponded to hexagonal graphite (COD # 96-101-1061). We attributed the observed widening of interplanar distances to three main reasons: (i) the noticeable increase in the amorphized phases; (ii) the bonding of oxygen and oxygen-containing carbon radicals along the edges of the {001} planes of hexagonal graphite and the penetration and non-covalent bonding of oxygen and oxygen-containing carbon radicals between these planes; (iii) the agglomeration of nano-sized phases during the drying of the samples. At present, we cannot clearly determine the influence of these factors. 

**Figure 13:**
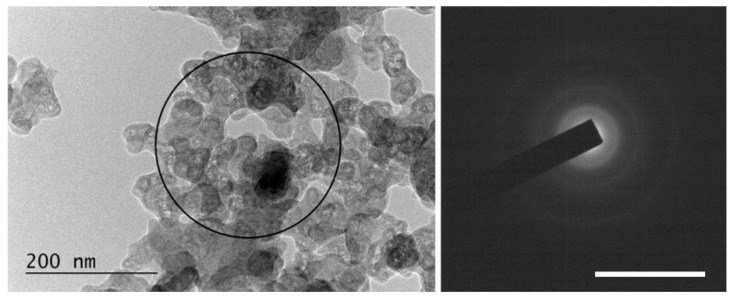
An overview image of completely amorphized graphitic carbon sedimented after irradiation of a suspension of HQ graphite in bidistilled water with a 355 nm laser. The scale bar on the SAED pattern corresponds to 10 1/nm.

- Relatively small (5–10 nm) 3D particles sedimented on the amorphous particles. These 3D particles were almost spherically shaped (Figure 12a) and possessed relatively clear SAED rings corresponding to the amorphous fullerene C_60_ (COD # 96-800-0216) (see Figure 12b), with HRTEM images showing inter-planar spacing consistent with fullerene C_60_ (COD # 96-800-0216) (see Figure 12c). Spherical particles that consisted of amorphous graphite were also observed. Such particles were more frequently observed irradiated by λ = 266 nm wavelength suspensions.
- Relatively large formations (size larger than 500 nm) with an irregular form and amorphous structure (see, for example, Figure 12d). Small single- to few-layered graphene flakes, as well as 3D nano-graphite particles (Figure 12f), were stuck to the surface of such amorphous particles (see Figure 12f). The corresponding SAED images (Figure 12e) contained reflections from disoriented structures of few-layered graphene and fullerene C_60_ (COD # 96-101-1061 and COD # 96-800-0216). These agglomerations were observed in HQ graphite suspensions in H_2_O irradiated with a λ = 266 nm wavelength only.

(iv) Practically completely amorphized graphitic carbon (see Figure 13). Such particles were observed in specimens from suspensions irradiated with a λ = 355 nm wavelength only.

## 4. Conclusions

This work investigates a proposed new method for PLAL in flow mode of an aqueous suspension of microcrystalline graphite, as well as in a flow of microcrystalline graphite, in a 6% H_2_O_2_ solution as a model system. It has been shown that irradiated suspensions containing mainly nanoscale carbon phases (graphene and graphene-like (defective graphene, rGO, and GO) and bundles of graphene-like flakes) can be produced by this method using laser-beam wavelengths of λ = 266 nm or 355 nm. The new approach for PLAL enables us to effectively achieve the desired degree of functionalization of the processed carbon materials with different types of atoms since the irradiation is carried out inside a quartz tube, isolated from the environment. The ability to control the medium, flow rate, laser wavelength, and irradiation energy flux enables the wide use of this technique. Finally, established flow-mode PLAL procedures have been found to be reproducible, and the main advantages of the proposed method are its simplicity, ease of application, and ability to synthesize large amounts of nano-sized carbon phases, as well as the opportunities it provides for effective functionalization.

## Figures and Tables

**Table 1:** Chemical composition of studied pristine and treated HQ and LQ graphite.

Sample	C, at.%	O, at.%	Sample	C, at.%	O, at.%
HQ pristine	97.96	2.04	LQ pristine	70.06	29.94
HQ+H_2_O, 266 nm	96.58	3.42	LQ+H_2_O 266 nm	68.57	31.43
HQ+H_2_O_2_ 266 nm	94.05	5.95	LQ+H_2_O_2_ 266 nm	61.73	38.27
HQ+H_2_O, 355 nm	97.55	2.45	LQ+H_2_O 355 nm	60.94	39.06
HQ+H_2_O_2_ 355 nm	96.02	3.98	LQ+H_2_O_2_ 355 nm	56.38	43.62

## Data Availability

Not applicable.
